# CO_2_ diffusion in tobacco: a link between mesophyll conductance and leaf anatomy

**DOI:** 10.1098/rsfs.2020.0040

**Published:** 2021-02-12

**Authors:** Victoria C. Clarke, Florence R. Danila, Susanne von Caemmerer

**Affiliations:** Australian Research Council Centre of Excellence for Translational Photosynthesis, Division of Plant Science, Research School of Biology, The Australian National University, Acton, Australian Capital Territory 2601, Australia

**Keywords:** CO_2_ diffusion, carbon isotope discrimination, cell wall, image analysis, leaf anatomy, leaf age

## Abstract

The partial pressure of CO_2_ at the sites of carboxylation within chloroplasts depends on the conductance to CO_2_ diffusion from intercellular airspace to the sites of carboxylation, termed mesophyll conductance (*g*_m_). We investigated how *g*_m_ varies with leaf age and through a tobacco (*Nicotiana tabacum*) canopy by combining gas exchange and carbon isotope measurements using tunable diode laser spectroscopy. We combined these measurements with the anatomical characterization of leaves. CO_2_ assimilation rate, *A*, and *g*_m_ decreased as leaves aged and moved lower in the canopy and were linearly correlated. This was accompanied by large anatomical changes including an increase in leaf thickness. Chloroplast surface area exposed to the intercellular airspace per unit leaf area (*S*_c_) also decreased lower in the canopy. Older leaves had thicker mesophyll cell walls and *g*_m_ was inversely proportional to cell wall thickness. We conclude that reduced *g*_m_ of older leaves lower in the canopy was associated with a reduction in *S*_c_ and a thickening of mesophyll cell walls.

## 1. Introduction

In plants with the C_3_ photosynthetic pathway, mesophyll conductance, *g*_m_, quantifies the ease with which CO_2_ diffuses from intercellular airspace within a leaf to the sites of Rubisco carboxylation within chloroplasts [1,2]. It is one of the three main physiological processes limiting CO_2_ uptake and fixation, the others being CO_2_ diffusion from the atmosphere to the sub-stomatal cavity (stomatal conductance, *g*_s_) and the biochemical activity of Rubisco and RuBP regeneration. Studies have shown that global crop production needs to double by 2050 to meet the projected demands from a rising population, diet shifts and increasing biofuels consumption [3]. The need to understand and maximize *g*_m_ is part of the research efforts being made to enhance photosynthesis to improve crop yield. For example, enhancements of photosynthesis through manipulation of chloroplast function will be diminished through the reduction in chloroplast CO_2_ partial pressure unless it is combined with improved *g*_m_ [4–9]. Increasingly, crop models are incorporating leaf and canopy level parameters to better predict where photosynthesis improvements can be made (e.g. [10]), and an understanding of mesophyll conductance variation across leaf positions is crucial for this.

**Figure 1.**
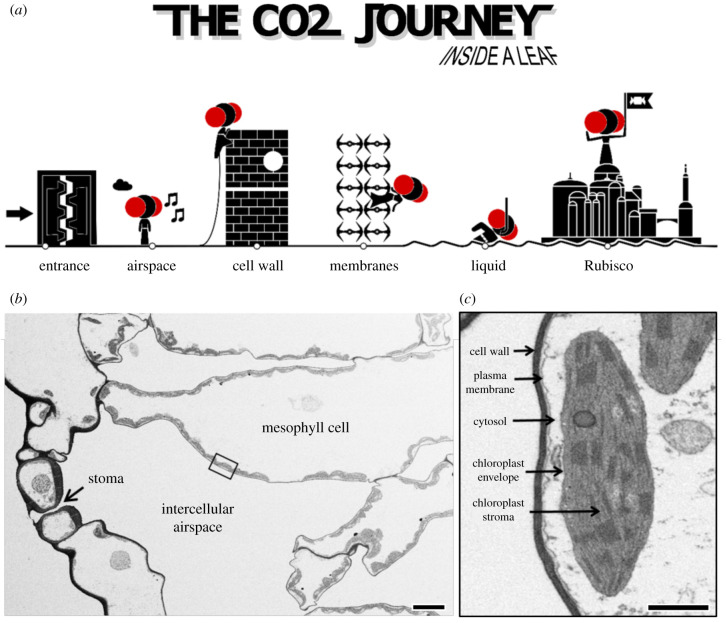
A Star Wars-inspired cartoon depicting the CO_2_ diffusion journey inside the leaf with Princess Leia representing a CO_2_ molecule (*a*) and the corresponding electron micrographs illustrating the actual CO_2_ diffusion pathway within a tobacco leaf (*b* and *c*). For photosynthetic CO_2_ assimilation to occur, CO_2_ has to diffuse through stomata (entrance), intercellular airspace, cell wall, membranes (plasma membrane and chloroplast envelope) and the liquid phase in the cytosol and chloroplast stroma. Once inside the chloroplast, CO_2_ is then fixed by Rubisco into energy-rich sugars as part of the photosynthesis process. Bar in *b* = 10 µm; bar in *c* = 1 µm.

At present, there is an incomplete mechanistic understanding of *g*_m_ (see Cousins *et al*. [11] for a review of recent developments in *g*_m_ in C_3_ and C_4_ species). To enhance CO_2_ diffusion, chloroplasts are spread thinly along cell wall surfaces, and their surface area appressing intercellular airspace is up to 20 times leaf surface area and good correlation between *g*_m_ and *S*_c_ (chloroplast surface area exposed to the intercellular airspace per unit leaf area) has been observed [12–14]. However, across species, this correlation is not unique and mesophyll cell wall thickness and its porosity, as well as membrane permeability to CO_2_ and liquid diffusion, are also considered important parameters of *g*_m_ [12,14,15]. Figure 1 outlines the CO_2_ diffusion path within the leaf.

There have been a number of studies that have examined variation in *g*_m_ with leaf age in tree species [14,16–18] but fewer studies have looked at variation in *g*_m_ with leaf age in crop species [19–21]. Here, we have examined the changes in CO_2_ assimilation rate and *g*_m_ with leaf age and canopy position in tobacco to assess the linkage between *g*_m_ and leaf anatomy, and provide information on how best to model variation in *g*_m_ in a C_3_ crop canopy.

## 2. Material and methods

### 2.1. Plant growth

Tobacco (*Nicotiana tabacum*, L. cv Samsun) was grown in a naturally lit glasshouse with day/night temperatures set at 28/18°C. Seeds were sown in the glasshouse in commercial seed raising mix, then transferred after two weeks to 5 l pots filled with commercial potting mix supplemented with slow-release fertilizer (Osmocote Exact, Scotts, NSW, Australia). Plants were grown in Canberra, Australia between June and August 2019, with an average day length of 10 h. Average light intensity at midday during the growing period was 700 µmol m^−2^ s^−1^. Plants were watered daily.

### 2.2. Concurrent measurements of gas exchange and carbon isotope discrimination to quantify mesophyll conductance

Gas exchange and carbon isotope discrimination measurements were made as described by Tazoe *et al*. [22] using a 6 cm^2^ chamber of the LI-6400 with a red–blue light-emitting diode (LED) light source (Li-Cor, Lincoln, NE, USA). Two LI-6400 chambers and the plants were placed in a temperature-controlled cabinet with fluorescent lights (TRIL1175, Thermoline Scientific Equipment, Smithfield, NSW 2164, Australia). The CO_2_ in the leaf chamber was set at 380 µmol mol^−1^, flow rate at 200 µmol s^−1^ and irradiance at 1500 µmol quanta m^−2^ s^−1^. Leaf temperature was controlled at 25°C. Two percent of O_2_ in N_2_, mixed by mass flow controllers (Omega Engineering Inc, Stamford, CT, USA), was supplied to the LI-6400s after humidification of the air by adjusting the temperature of the water circulating around a Nafion tube (Perma Pure LLC, Toms River, NJ, USA, MH-110-12P-4). Gas exchange was coupled to a tunable diode laser (TDL; TGA100a, Campbell Scientific, Inc., Logan, UT, USA) for concurrent measurements of carbon isotope composition. Measurements were made at 4 min intervals for 20 s, with 10–12 measurements per leaf and the last five measurements were averaged. The *δ*^13^C of CO_2_ gas cylinders (*δ*^13^C_tank_) used in the LI-6400 CO_2_ injector system was 14‰. Gas exchange was calculated using the equations presented by von Caemmerer and Farquhar [23] and *Δ* was calculated from the equation presented by Evans *et al*. [24]. Values of *ξ* = *C*_ref_/(*C*_ref_ − *C*_sam_) ranged between 4 and 14, where *C*_ref_ and *C*_sam_ are the CO_2_ concentrations of dry air entering and exiting the leaf chamber, respectively, measured by the TDL. Measurements were taken on four 6- to 9-week-old plants with 3–12 leaves. Mesophyll conductance, *g*_m_, was calculated as described by Evans & von Caemmerer [25].

### 2.3. Biochemical measurements of Rubisco site content and leaf nitrogen

Total Rubisco content was estimated from leaf discs by the irreversible binding of [^14^C]2-carboxy-D-arabinitol 1,5-bisphosphate to the fully carbamylated enzyme as described by Ruuska *et al*. [26]. Leaf nitrogen (N) was determined on leaf discs which were oven-dried at 80°C, weighed and then ground to powder. Percentage of N was determined on the ground tissues using a flash combustion CNS analyser (Fison NA1500; Fison Instruments, Milan, Italy).

### 2.4. Anatomical measurements

Leaf anatomy was determined from light and scanning electron micrographs of transverse sections of resin-embedded leaf tissue collected from leaf positions 1 to 10 of three 9-week-old tobacco plants (figure 3). Leaf tissue was collected from the same area used for the concurrent gas exchange and carbon isotope discrimination measurement and immediately processed for light and electron microscopy as previously described [27]. Light micrographs were obtained using a Leica DM5500 compound microscope (Leica Microsystems) and used to measure leaf thickness and mesophyll layer thickness. For scanning electron microscopy, ultrathin sections were mounted onto pieces of silicon wafer and post-stained with aqueous uranyl acetate followed by lead citrate for 10 min each prior to imaging under a Zeiss UltraPlus field emission scanning electron microscope at 2 kV. Scanning electron micrographs were used to measure mesophyll cell wall thickness, chloroplast length, chloroplast thickness, the surface area of mesophyll cells exposed to intercellular airspace per unit leaf area (*S*_mes_) and surface area of chloroplasts exposed to intercellular airspace per unit leaf area (*S*_c_). Electron micrograph measurements were performed according to Evans *et al*. [13] using at least 600 µm leaf surface length for each leaf position per plant. Stomatal density was measured from positives made with nail polish from hydrophilic vinyl polysiloxane impressions of the abaxial surface of the leaf area measured by gas exchange, and viewed under a Leica confocal microscope and Leica DC500 camera. All measurements were made using ImageJ software (National Institutes of Health) and a Wacom Cintiq graphics tablet (Wacom Technology).

### 2.5. Statistical analysis

**Figure 2.**
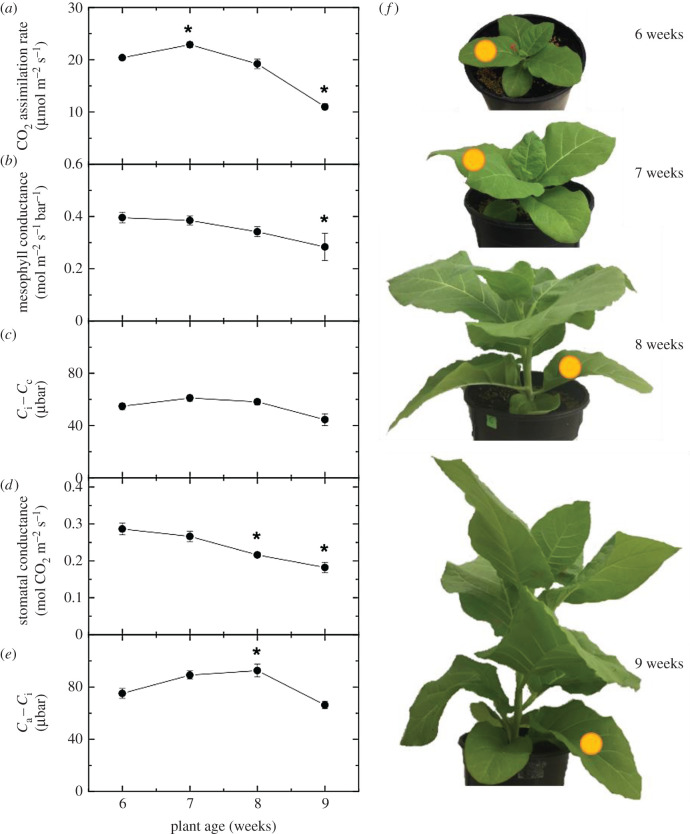
Variation in CO_2_ assimilation rate, mesophyll conductance, *C*_i_ − *C*_c_, stomatal conductance and *C*_a_ − *C*_i_ over time with increasing leaf age. Gas exchange measurements were made at an irradiance of 1500 µmol m^−2^ s^−1^, ambient CO_2_ of 380 µbar, 2% O_2_ and a leaf temperature of 25°C. Gas exchange measurements were made concurrently with measurements of carbon isotope discrimination using tunable diode laser spectroscopy for the calculation of mesophyll conductance (see Materials and methods), *n* = 4. Timepoints significantly different from 6-week-old plants are indicated with an asterisk. The same leaves were followed over time, and measured leaf is denoted with a yellow circle on plant images. Raw data are given in the electronic supplementary material, Data File S1.

Statistical analyses were performed using Student's *t*-test for figure 2, and all other data using one-way analysis of variance. Means comparison was made at 0.05 significance level using the Tukey test (OriginPro 2020, OriginLab Corporation).

## 3. Results

### 3.1. Changes in leaf physiology over time

To understand how mesophyll conductance, *g*_m_, is influenced by leaf ageing, leaf physiology traits were repeatedly measured in a tobacco leaf over four weeks of growth. CO_2_ assimilation rate, *A*, stomatal conductance, *g*_s_, and *g*_m_ all decreased in the ageing tobacco leaf over time (figure 2). The drawdown of CO_2_ into the chloroplast (*C*_i_ − *C*_c_), however, was not significantly changed over the four weeks of measurements. The drawdown of CO_2_ into the sub-stomatal cavity (*C*_a_ − *C*_i_) increased between 6 and 8-week-old leaves, but was not significantly different overall between the 6-week and 9-week measurements (figure 2). Raw data for figure 2 are given in the electronic supplementary material, Data File S1.

### 3.2. The effect of canopy position on leaf anatomy and physiology

**Figure 3.**
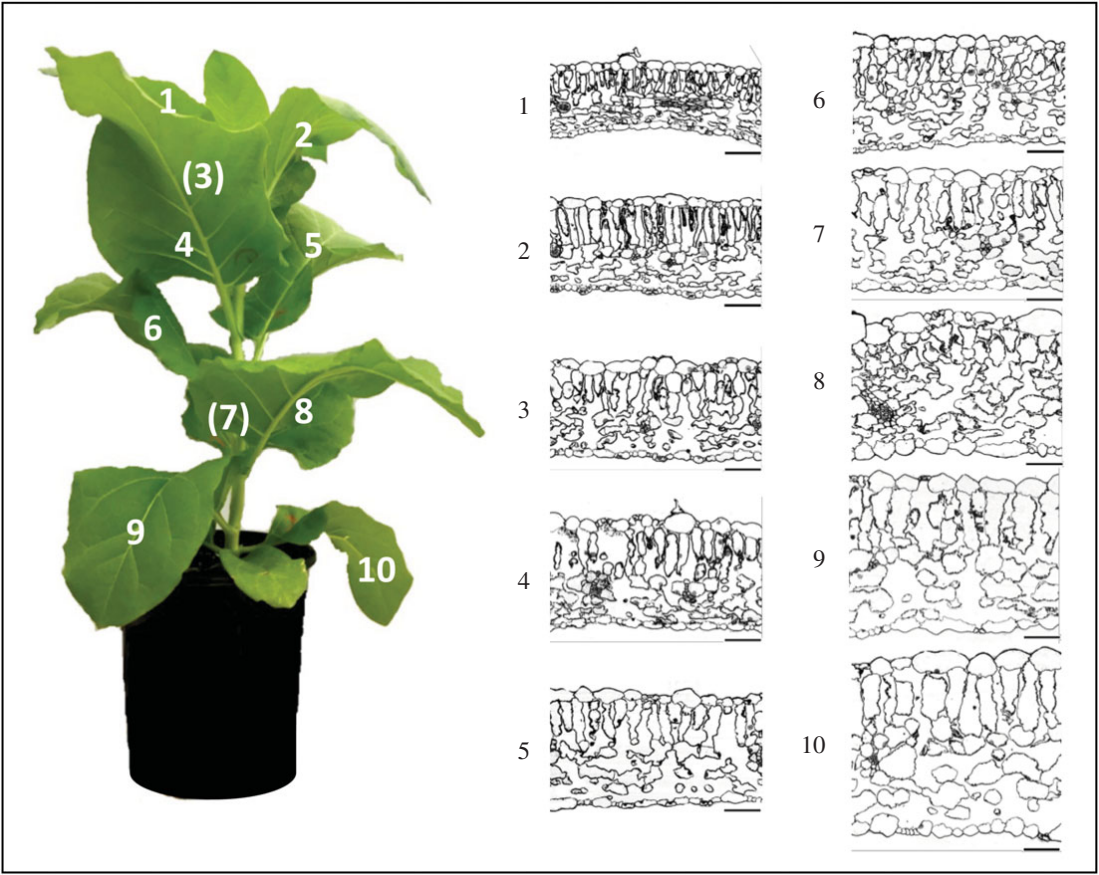
Light micrographs of transverse sections of resin-embedded leaf tissue collected from leaf position 1 to 10 of 9-week-old tobacco plant showing variation in leaf thickness (see figure 5*b* for measured values). Bars = 100 µm.

**Figure 4.**
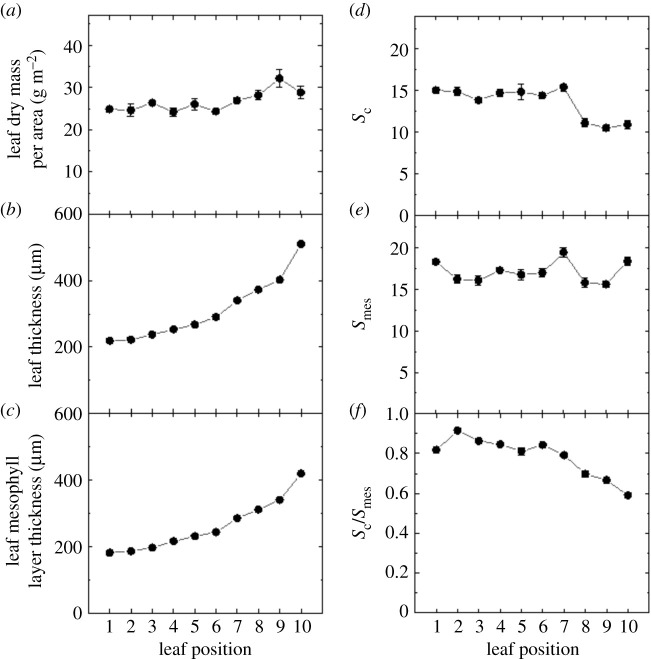
Variation in leaf dry mass per area (LMA, *n* = 4 plants) (*a*), leaf thickness (*b*), leaf mesophyll layer thickness (*c*), *S*_c_, chloroplast surface area exposed to intercellular airspace per unit leaf area (*d*), *S*_mes_, mesophyll surface area exposed to intercellular airspace per unit leaf area (*e*), and *S*_c_/*S*_mes_ (*f*) with leaf position in the canopy (figure 3). For anatomical measurements, *n* = 3 plants. Raw data are given in the electronic supplementary material, Data File S2.

**Figure 5.**
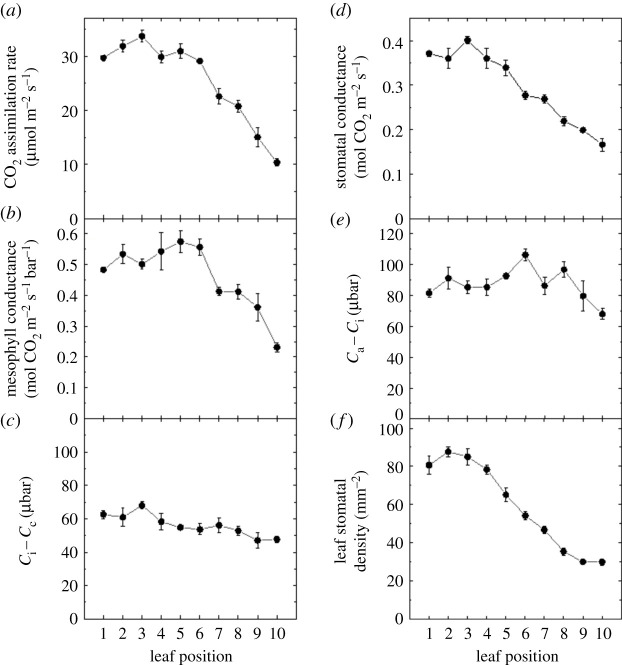
Variation in CO_2_ assimilation rate (*a*), mesophyll conductance (*b*), *C*_i_ − *C*_c_ (*c*), stomatal conductance (*d*), *C*_a_ − *C*_i_ (*e*) and abaxial stomatal number per leaf area (*f*) with leaf position in the canopy (figure 3). Gas exchange measurements were made at an irradiance of 1500 µmol m^−2^ s^−1^, ambient CO_2_ of 380 µbar, 2% O_2_ and a leaf temperature of 25°C. Gas exchange measurements were made concurrently with measurements of carbon isotope discrimination using tunable diode laser spectroscopy for the calculation of mesophyll conductance (see Materials and methods), *n* = 4 plants. Raw data are given in the electronic supplementary material, Data File S2.

**Figure 6.**
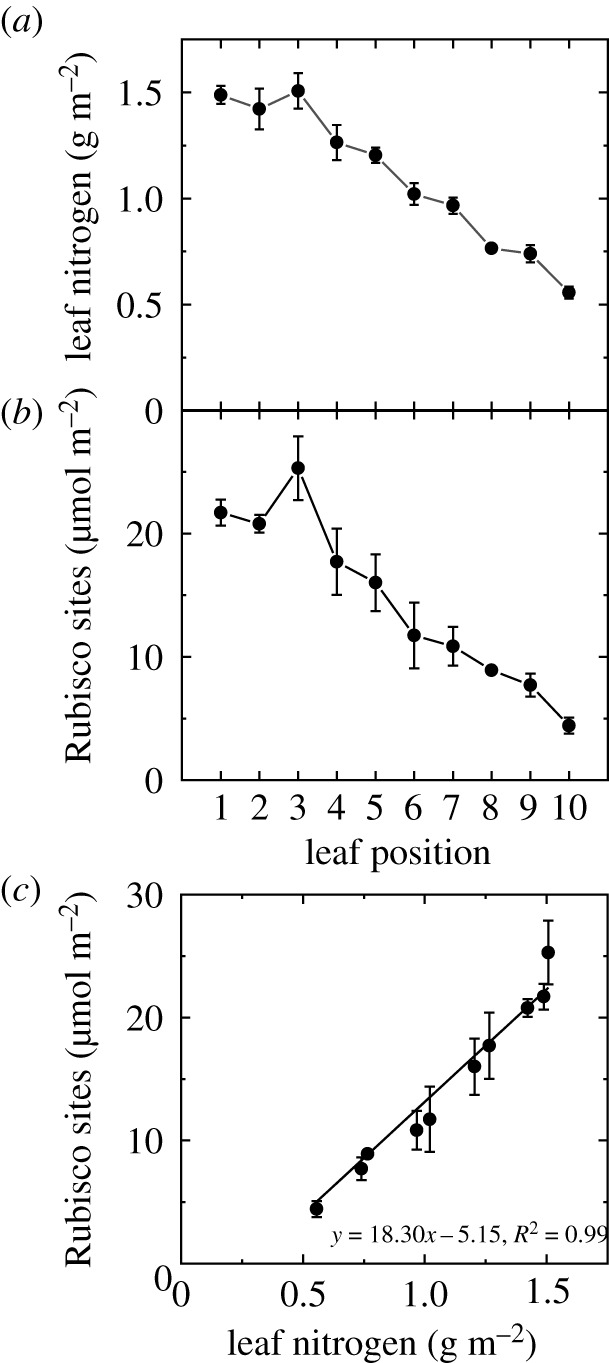
Variation in leaf nitrogen (*a*) and Rubisco site content (*b*) with leaf position in the canopy (figure 3). The relationship between Rubisco sites and leaf nitrogen is shown in (*c*), *n* = 4 plants. Raw data are given in the electronic supplementary material, Data File S2.

The effect of leaf canopy position was further investigated in 9-week-old tobacco plants with 10 leaves through measurement of physiological and anatomical traits. To determine whether there is a direct correlation between the leaf anatomy and *g*_m_, structural and ultrastructural analyses were performed using the leaf tissue from the same area as used for physiological measurement. Light micrographs of transverse leaf sections showed that leaf thickness and mesophyll cell layer thickness increase as the leaf matures (figures 3 and 4*b*,*c*). Surprisingly this was not accompanied by a significant increase in leaf mass per area (LMA, figure 4*a*), which varied from 24.8 ± 0.6 g m^−2^ in leaf 1 to 32.1 ± 2.1 g m^−2^ in leaf 9. Measurements performed using electron micrographs of the same leaf sections revealed that chloroplast surface area exposed to the intercellular airspace per unit leaf area (*S*_c_, figure 4*d*) and mesophyll surface area exposed to the intercellular airspace per unit leaf area (*S*_mes_, figure 4*e*) decrease after leaf position 6. Reduced values of *S*_c_ and *S*_mes_ in leaf positions 7–10 (figure 4*d,e*) resulted in decreasing values of chloroplast cover of the exposed mesophyll cells, *S*_c_/*S*_mes_, in the same leaf positions (figure 4*f*). Significant statistical differences of means at the 5% level (*p* < 0.05) are summarized in table 1 for all parameters shown in figures 4–6 and 8, and the raw data are given in the electronic supplementary material, Data File S2.

**Table 1.** Means comparison using Tukey test of the physiological and anatomical parameters measured between different leaf positions of tobacco plant. The number 1 denotes significant difference at *p* < 0.05 and 0 denotes no significant difference at *p* < 0.05.

leaves compared	*A*	*C*_a_ − *C*_i_	*C*_i_ − *C*_c_	*g*_m_	*g*_s_	stomatal density	leaf N	Rubisco sites	LMA	leaf thickness	leaf M layer thickness	*S*_c_	*S*_mes_	*S*_c_/*S*_mes_	chloroplast thickness	chloroplast length	cell wall thickness
2 1	0	0	0	0	﻿0	0	0	0	0	0	0	0	0	1	1	0	0
3 1	0	0	0	0	0	0	0	0	0	0	1	0	1	0	1	0	1
3 2	0	0	0	0	0	0	0	0	0	0	1	0	0	0	0	0	1
4 1	0	0	0	0	0	0	0	0	0	1	1	0	0	0	1	0	1
4 2	0	0	0	0	0	0	0	0	0	1	1	0	0	1	0	0	1
4 3	0	0	0	0	0	0	0	0	0	0	1	0	0	0	0	0	0
5 1	0	0	0	0	0	1	1	0	0	1	1	0	0	0	1	0	0
5 2	0	0	0	0	0	1	0	0	0	1	1	0	0	1	0	0	0
5 3	0	0	0	0	0	1	1	1	0	1	1	0	0	0	0	0	1
5 4	0	0	0	0	0	0	0	0	0	0	1	0	0	0	0	0	1
6 1	0	0	0	0	1	1	1	1	0	1	1	0	0	0	1	1	1
6 2	0	0	0	0	1	1	1	1	0	1	1	0	0	1	1	0	1
6 3	0	0	0	0	1	1	1	1	0	1	1	0	0	0	1	1	0
6 4	0	0	0	0	1	1	0	0	0	1	1	0	0	0	1	1	0
6 5	0	0	0	0	0	0	0	0	0	0	1	0	0	0	1	1	1
7 1	1	0	0	0	1	1	1	1	0	1	1	0	0	0	1	0	1
7 2	1	0	0	0	1	1	1	1	0	1	1	0	1	1	0	0	1
7 3	1	0	0	0	1	1	1	1	0	1	1	0	1	1	0	0	1
7 4	1	0	0	0	1	1	1	0	0	1	1	0	1	1	0	0	1
7 5	1	0	0	1	1	1	0	0	0	1	1	0	1	0	0	0	1
7 6	1	0	0	0	0	0	0	0	0	1	1	0	1	0	1	1	1
8 1	1	0	0	0	1	1	1	1	0	1	1	1	1	1	1	0	1
8 2	1	0	0	0	1	1	1	1	0	1	1	1	0	1	0	0	1
8 3	1	0	0	0	1	1	1	1	0	1	1	1	0	1	1	0	1
8 4	1	0	0	0	1	1	1	0	0	1	1	1	0	1	1	0	1
8 5	1	0	0	1	1	1	1	0	0	1	1	1	0	1	0	0	1
8 6	1	0	0	0	0	1	0	0	0	1	1	1	0	1	1	0	1
8 7	0	0	0	0	0	0	0	0	0	1	1	1	1	1	0	0	1
9 1	1	0	0	0	1	1	1	1	1	1	1	1	1	1	0	1	1
9 2	1	0	0	1	1	1	1	1	1	1	1	1	0	1	1	1	1
9 3	1	0	1	0	1	1	1	1	1	1	1	1	0	1	1	1	1
9 4	1	0	0	1	1	1	1	1	1	1	1	1	0	1	1	1	1
9 5	1	0	0	1	1	1	1	0	1	1	1	1	0	1	1	1	1
9 6	1	1	0	1	1	1	1	0	1	1	1	1	0	1	1	0	1
9 7	1	0	0	0	1	1	0	0	0	1	1	1	1	1	1	1	1
9 8	1	0	0	0	0	0	0	0	0	1	1	0	0	0	1	1	1
10 1	1	0	0	1	1	1	1	1	0	1	1	1	0	1	1	1	1
10 2	1	0	0	1	1	1	1	1	0	1	1	1	0	1	0	1	1
10 3	1	0	1	1	1	1	1	1	0	1	1	1	0	1	1	1	1
10 4	1	0	0	1	1	1	1	1	0	1	1	1	0	1	1	1	1
10 5	1	0	0	1	1	1	1	1	0	1	1	1	0	1	0	1	1
10 6	1	1	0	1	1	1	1	0	0	1	1	1	0	1	1	1	1
10 7	1	0	0	1	1	1	1	0	0	1	1	1	0	1	1	1	1
10 8	1	1	0	1	0	0	0	0	0	1	1	0	1	1	0	1	1

CO_2_ assimilation rate, *A*, decreased from maximal values in leaves at the top of the leaf canopy down to leaves at the base of the canopy, particularly from leaf 7–10 (figure 5*a*,*b*, table 1). Mesophyll conductance decreased strongly from leaf 6 onwards. Stomatal conductance also showed a similar decrease in the canopy (figure 5*d*). The variation in the drawdown of CO_2_ from the atmosphere into the sub-stomatal cavity (*C*_a_ − *C*_i_, figure 5*e*) and from the sub-stomatal cavity into the chloroplasts (*C*_i_ − *C*_c_, figure 5*c*) was less dynamic, but significant differences were still apparent between leaves at the top and bottom of the canopy (table 1). The number of stomata per mm^2^ leaf area (stomatal density, figure 5*f*) decreased moving down the canopy due to the overall expansion of the pavement cells on the leaf surface as the leaves aged (electronic supplementary material, figure S1). There was a strong correlation between stomatal conductance and stomatal density (*y* = 0.0034*x* + 0.092, *R^2^* = 0.95).

Leaf nitrogen (N) content was highest in the youngest leaves at the top of the canopy, and steadily declined through the lower leaf positions (figure 6*a*). Rubisco content also decreased down the canopy (figure 6*b*) and was found to be very closely correlated to leaf nitrogen content (figure 6*c*, *R^2^* = 0.99). There was a strong linear relationship between *g*_m_ and *A* (*y* = 0.0126*x* + 0.142, *R^2^* = 0.71, electronic supplementary material, figure S2). However, the relationships between *g*_m_ and Rubisco content or leaf N were best fitted with a second-order polynomial (electronic supplementary material, figure S2) as were the relationships between *A* and Rubisco content and leaf N (electronic supplementary material, figure S3). This shows that *A* per Rubisco or leaf N was less in leaves at the top of the canopy than in older leaves further down the canopy.

**Figure 7.**
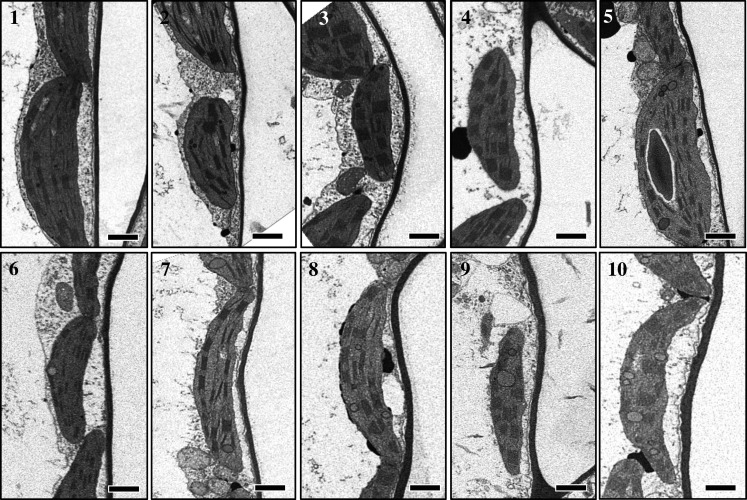
Electron micrographs of transverse sections of resin-embedded leaf tissue collected from leaf position 1 to 10 of 9-week-old tobacco plant showing variation in mesophyll cell wall thickness (see figure 8*c* for measured values). Bars = 1 µm.

**Figure 8.**
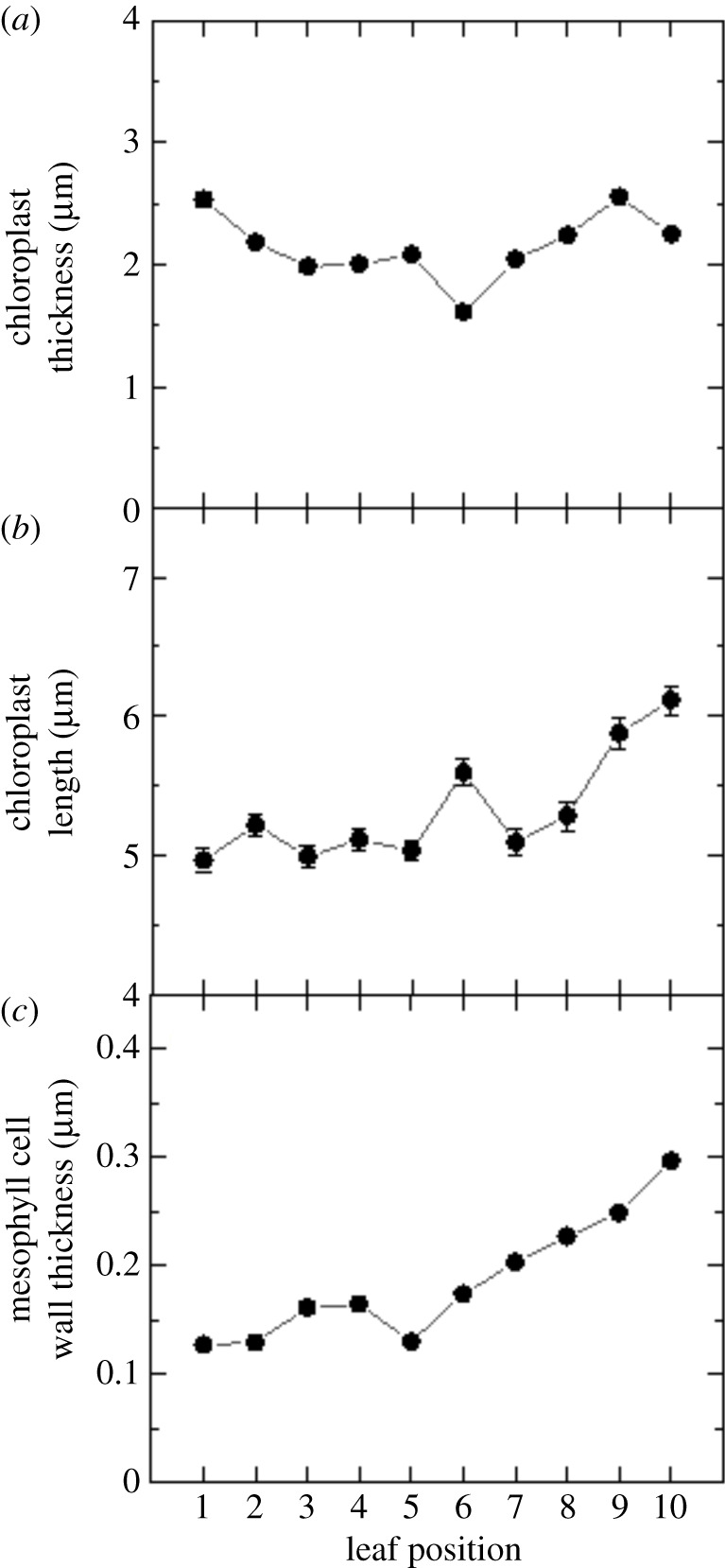
Variation in chloroplast thickness (*a*), chloroplast length (*b*) and mesophyll cell wall thickness (*c*) with leaf position in the canopy (figure 3), *n* = 3 plants. Raw data are given in the electronic supplementary material, Data File S2.

**Figure 9.**
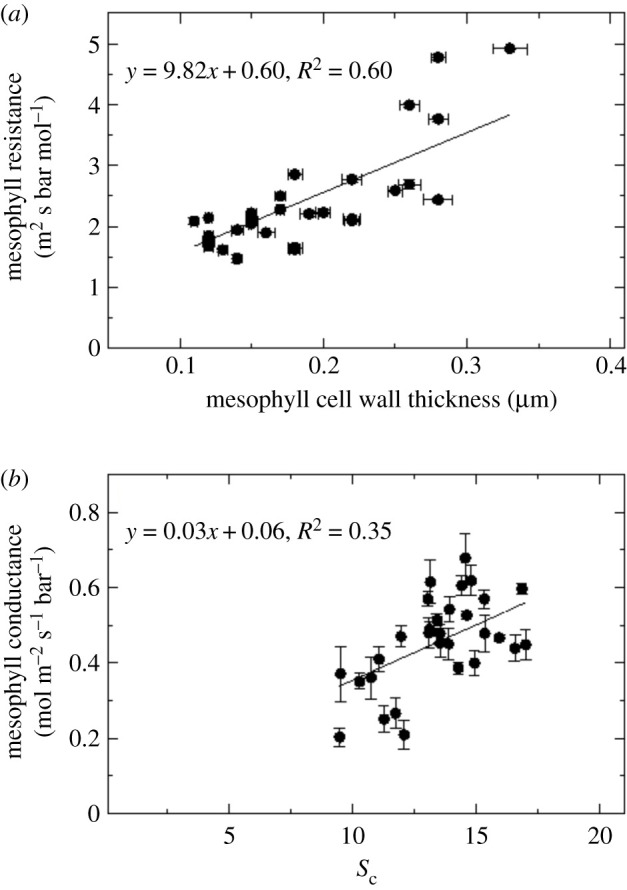
The relationship between mesophyll resistance (*r*_m_ = 1/*g*_m_) and mesophyll cell wall thickness (*a*) and between mesophyll conductance and *S*_c_, the chloroplast surface area exposed to intercellular airspace per unit leaf area (*b*). Each data point corresponds to an average value per leaf from three plants. Raw data are given in the electronic supplementary material, Data File S2.

Electron micrographs also allowed measurements of mesophyll chloroplast thickness, mesophyll chloroplast length and mesophyll cell wall thickness in leaf positions 1–10 (figure 7). Results showed a decrease in mesophyll chloroplast thickness (figure 8*a*), an increase in mesophyll chloroplast length (figure 8*b*) and thickening of mesophyll cell walls (figure 8*c*) as the leaf matures. Measurements and calculations also revealed that the thicker mesophyll cell wall in older leaves (figure 8*c*) was directly proportional to the mesophyll resistance (*r*_m_ = 1/*g*_m_, figure 9*a*). This resistance ranged from 1.5 in young leaves to 4 m^2^ s bar mol^−1^ in lower canopy leaves and the inverse *g*_m_ ranged from 0.7 to 0.2 mol m^−2^ s^−1^ bar^−1^. There was only a weak correlation between mesophyll conductance and the chloroplast surface area exposed to intercellular airspace (*S*_c_, figure 9*b*).

## 4. Discussion

### 4.1. Mesophyll conductance correlates with CO_2_ assimilation rate

CO_2_ assimilation rate, *A*, and mesophyll conductance, *g*_m_, both decreased as leaves moved lower in the tobacco canopy, with a strong linear correlation between *A* and *g*_m_ reflected in a constant difference in CO_2_ partial pressure between intercellular airspace and the chloroplasts (*C*_i_ − *C*_c_) across leaf positions (figure 5, and electronic supplementary material, figure S2). Strong correlations between *A* and *g*_m_ have previously been observed with leaf development or ageing [14,16,19,20,28,29]. In a study with transgenic tobacco plants where Rubisco content was reduced, the correlation did not hold and *A* declined more than *g*_m_ [13]. This indicates that it is not a mechanistic relationship between *A* and *g*_m_. Both *A* and *g*_m_ showed curvilinear responses to Rubisco and leaf nitrogen (electronic supplementary material, figures S2 and S3) suggesting that the link between *A* and *g*_m_ is the most useful for the incorporation of *g*_m_ in canopy photosynthesis and crop models [10,30–32].

### 4.2. Relationship between CO_2_ assimilation rate and Rubisco and leaf N content

Rubisco accounts for around 40% of soluble protein in a leaf and around 20% of leaf nitrogen (N) investment in C_3_ species [33]. We saw a very close correlation between leaf N and Rubisco, suggesting that the proportional investment in Rubisco across leaves remained constant across the canopy. We also observed curvilinear relationships between *A* and Rubisco content or leaf N (electronic supplementary material, figure S3) with young leaves having a lower Rubisco and leaf N use efficiency. Curvilinear responses have been observed in the past and two explanations have been put forward [34]. The first one suggests it could be a CO_2_ diffusion limitation, but since we know that there has been no increase in either *C*_a_ − *C*_i_ or *C*_i_ − *C*_c_ we can rule out a CO_2_ diffusion limitation. The second reason given by Evans [34] is that with increasing leaf N the chlorophyll and electron transport capacity increase and to reach light saturation requires progressively higher irradiances. Since we measured at the same irradiance the maximal rate of the high nitrogen leaf is underestimated. We only made measurements of *A* at one irradiance and this could be further investigated.

### 4.3. Linking mesophyll conductance and leaf anatomy

Figures 3, 4 and 7–9 highlight the large anatomical changes that occur during leaf development. There was a doubling of leaf thickness but this did not result in significant changes in leaf mass per area (LMA) despite the increase in cell wall thickness. In an across species comparison higher LMA has been associated with decreased *g*_m_ and an increase in the percentage that cell wall mass contributes to LMA [35]. However, tobacco, as a herbaceous crop species, has a low LMA compared to species tested in that study, and cell wall mass is expected to contribute no more than 10–15% of LMA (figure 3; [35]). It is therefore not surprising that we saw little change in LMA despite an increase in cell wall thickness and reduction in *g*_m_. We conclude that increased airspace and cell volume account for these changes.

To facilitate CO_2_ diffusion, chloroplasts line mesophyll cell walls adjacent to intercellular airspace. Anatomical parameters such as *S*_c_ and *S*_mes_ are similar to those measured in glasshouse grown tobacco in previous studies [13] providing a chloroplast surface area 15 times greater than the projected leaf area. As chloroplasts cover 80% or more of mesophyll cell walls adjacent to intercellular airspace, there is room for only small improvements of *g*_m_ by increasing *S*_c_/*S*_mes_. However, *S*_c_/*S*_mes_ has been shown to be less in low light conditions and under stress in *Populus tremula*, and can vary with growth conditions [14]. Compared to the rapid decline in CO_2_ assimilation rate with leaf position, *S*_c_ remained unchanged up to leaf position 7 and is therefore not the driver for the reduction in CO_2_ assimilation rates or *g*_m_ (figure 9*b*). Other studies that have also compared changes in *A*, *g*_m_ and *S*_c_ with leaf age variation also reported the poor correlation between *g*_m_ and *S*_c_ under these conditions [16,19,20].

As depicted in figure 1 CO_2_ has to diffuse from intercellular airspace across the cell wall, the plasma membrane, cytosol, chloroplast envelope and the chloroplast stroma. Of these obstacles in the diffusion path, cell wall thickness and chloroplast shape are measurable components. We observed a continuous increase in cell wall thickness from the top to the bottom of the canopy and observed a strong inverse correlation between *g*_m_ and cell wall thickness (figure 9*a*). This highlights the important contribution of cell wall thickness in determining *g*_m_ and is in line with previous studies [12,15,36,37]. Tobacco like many crop species has relatively thin cell walls [36]; however, it is not just the physical dimension but also cell wall composition that matters and we know little about this at present (see [38] for a review of current knowledge). If we extrapolate to zero cell wall thickness we see a resistance of 0.6 m^2^ s bar mol^−1^ (figure 9*a*) equating to a mesophyll conductance of 1.6 mol m^−2^ s^−1^ bar^−1^. While we cannot accurately measure the resistance derived from plant membranes, we know that membrane composition (such as the presence of channels for facilitating CO_2_ transfer) together with CO_2_ diffusion through the liquid phases also contributes to mesophyll resistance [39]. Nevertheless, our results highlight that thinner cell walls, if structurally possible, could be of benefit to improve mesophyll conductance.

## 5. Conclusion

We combined gas exchange and anatomical measurements to assess how CO_2_ assimilation rate, *A*, and mesophyll conductance, *g*_m_, decreased as leaves aged and whether we could link decreases in *g*_m_ with leaf anatomy. Surprisingly we observed a decrease in the chloroplast surface area exposed to the intercellular airspace per unit leaf area, *S*_c_, only low in the canopy whereas there was a gradual increase in cell wall thickness and an inverse correlation between *g*_m_ and cell wall thickness. We conclude that reduced *g*_m_ of older leaves lower in the canopy was associated with a reduction in *S*_c_ and a thickening of mesophyll cell walls. The relationship between *A* and *g*_m_, however, is the most useful for the incorporation of *g*_m_ in canopy photosynthesis and crop models. In crop species where mesophyll cell chloroplast cover is high, increasing the conductance across the chloroplast interface is the major challenge for improvements in mesophyll conductance, which will enhance photosynthetic capacity and ultimately increase crop yields.

## Supplementary Material

Supplementary Data File 1

## Supplementary Material

Supplementary Data File 2

## Supplementary Material

Supplementary Figures

## Data accessibility

Raw data are available in the electronic supplementary materials of this article.
