# Supplementary material for: CO2 diffusion in tobacco: a link between mesophyll conductance and leaf anatomy
Source: Interface Focus. 2021 Feb 12;11(2):20200040. doi: 10.1098/rsfs.2020.0040 (PMC7898150; doi:10.1098/rsfs.2020.0040)
Supplement: Supplementary Figures [file rsfs20200040supp3.pdf]

Supplementary Figures
Clarke, Danila and von Caemmerer "CO_2 diffusion in tobacco: a link between mesophyll conductance and leaf anatomy" *Interface Focus*.

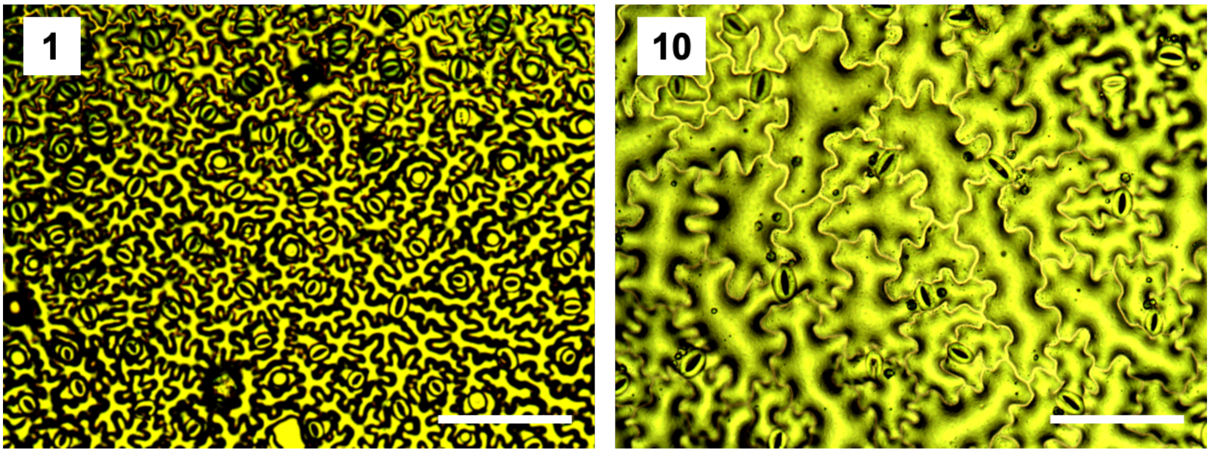

Supplementary Figure S1

Leaf surface imprints of leaf position 1 and 10 of 9-week old tobacco plants, showing variation in stomatal density (see Figure 6f for measured values). Bars = 200 µm.

Supplementary Figures
Clarke, Danila and von Caemmerer "CO2 diffusion in tobacco: a link between mesophyll conductance and leaf anatomy" *Interface Focus*.

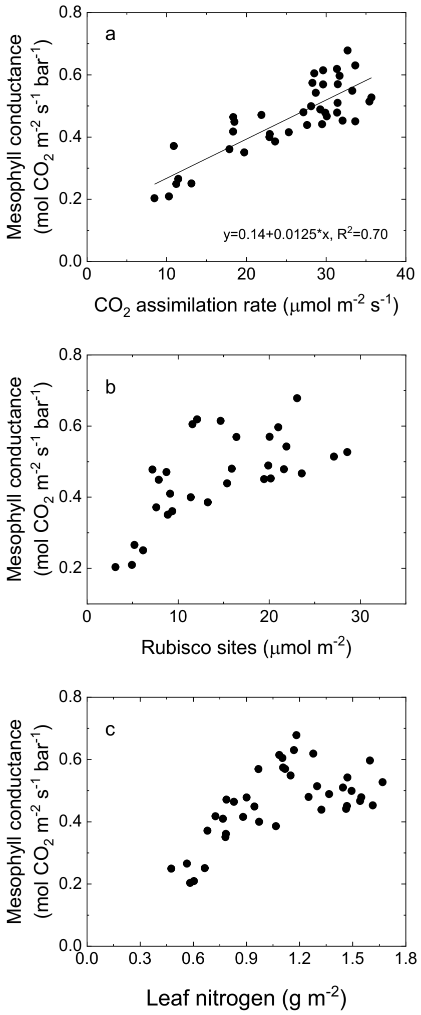

Supplementary Figure S2

Mesophyll conductance as a function of CO_2 assimilation rate (a), Rubisco sites (b) or Leaf nitrogen content (c).

Supplementary Figures
Clarke, Danila and von Caemmerer "CO2 diffusion in tobacco: a link between mesophyll conductance and leaf anatomy" *Interface Focus*.

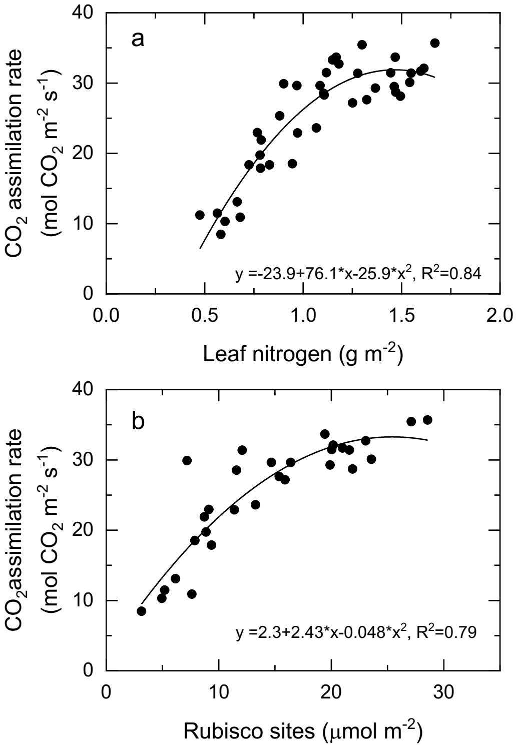

Supplementary Figure S3

CO_2 assimilation rate as a function of leaf nitrogen (a) and Rubisco site content (b).
